# Obituary: Professor Maurizio Bonati

**DOI:** 10.1136/bmjpo-2026-005471

**Published:** 2026-10-05

**Authors:** Imti Choonara

**Affiliations:** 1 University of Nottingham School of Medicine, Derby, UK

**Keywords:** Pharmacology

*BMJ Paediatrics Open* is saddened to hear of the sudden death of Maurizio Bonati at the age of 72.

Maurizio played a key role in supporting the journal as an active member of the Editorial Advisory Board. He was a regular reviewer and contributor to the journal.

He was one of the leading paediatric clinical pharmacologists in the world. Early work focused on the epidemiology of the use of medicines and surveillance of drug toxicity in children. He was the first to recognise the importance of transparency in paediatric clinical trials and established the European Registry of Clinical Trials in Children (DEC-net) in 2004. This was the first population-specific clinical trials registry and was coordinated by Maurizio from the Mario Negri Institute in Milan, where he was Director of the Laboratory for Maternal and Child Health. Despite being ahead of most of his peers academically, he remained modest in his manner and the importance of his work.

He was involved in research looking at the extent of off-label drug use in children which resulted in European legislation focusing on clinical trials in children. He recognised the importance of treatment being evidence-based and that evidence should be transparent and open to parents as well as health professionals.^1^ He was an expert in systematic reviews and meta-analyses and evaluated the efficacy of selective serotonin reuptake inhibitors in childhood depression.

He published extensively and focused on the diagnosis and treatment of attention deficit hyperactivity disorder (ADHD) in children. He was instrumental in establishing the ADHD Register in the Lombardy Region of Italy. He expanded his research interest from paediatric clinical pharmacology to public health and subsequently became Director of Public Health at the Mario Negri Institute.

He also studied the transition process from paediatric to adult care for chronic diseases, in particular ADHD, diabetes and epilepsy. His research was always focused on improving clinical care for children and their families.

Alongside collaborating with others in Europe, he was interested in global child health. He encouraged research in child health in Latin America and supported the Bienal de Pediatria in Colombia (a biannual event evaluating child health research from Latin America). He collaborated with researchers in countries from Africa, Europe and Latin America.

The breadth of his research ranged from evaluating the adverse psychological effects of lockdown (quarantine) on Italians during the COVID epidemic to the extent of obesity in Italian children.^2^ Some of the other areas that he published in included the management of tuberculosis in pregnancy; screening for tuberculosis in migrants; home ventilation in children; safety in clinical trials; nasal continuous positive airway pressure in low income countries.

After retirement from the Mario Negri Institute, he took on the role of Editor of the Italian journal *Ricerca & Pratica* (Research & Practice).

He recognised the devastating effects of war on children and published a book (*Il cronico trauma del guerra*) on the effects of war on children. He also published several articles in *BMJ Paediatrics Open* on the conflict in Gaza. This reflected his love for humanity. He is survived by his daughter and grandson, whom he visited regularly.
